# Neurosarcoidosis and Transverse Myelitis: Life-Threatening Manifestations of Sarcoidosis

**DOI:** 10.7759/cureus.52629

**Published:** 2024-01-20

**Authors:** Mitwa Patel, Sheena Shiwlani, Meet Popatbhai Kachhadia, Mohamed Abdalla, Iqra Samreen, Alaa S Mohamed, Hira Nasir

**Affiliations:** 1 Internal Medicine, David Tvildiani Medical University, Tbilisi, GEO; 2 Pathology, Mount Sinai Hospital, New York, USA; 3 Internal Medicine, Pandit Dindayal Upadhyay (PDU) College, Civil Hospital Campus, Rajkot, IND; 4 Internal Medicine, Dallah Hospital, Riyadh, SAU; 5 Internal Medicine, Augusta University, Augusta, USA; 6 Neurology, Augusta University, Augusta, USA; 7 Internal Medicine, Mayo Hospital, Lahore, PAK

**Keywords:** transverse myelitis, non-caseating granulomas, longitudinally extensive transverse myelitis, neurosarcoidosis, sarcoidosis

## Abstract

Sarcoidosis, a systemic granulomatous disorder, typically involves the lungs, skin, and lymph nodes. Neurological manifestations are diverse and may include longitudinally extensive transverse myelitis (LETM), an uncommon inflammatory disorder of the spinal cord. We present a case of a 62-year-old female with LETM as the initial manifestation of sarcoidosis. The patient exhibited progressive bilateral lower extremity weakness, urinary retention, and sensory disturbances. Diagnostic workup revealed characteristic findings on spinal magnetic resonance imaging (MRI), cerebrospinal fluid analysis, and thoracic biopsy. Treatment with high-dose corticosteroids and subsequent immunomodulatory therapy resulted in significant improvement. Our case highlights the importance of including sarcoidosis in the differentials of LETM, particularly in patients with no respiratory manifestations.

## Introduction

Sarcoidosis, a systemic granulomatous disorder, is characterized by granulomas in response to abnormal collections of inflammatory cells, impacting various organs and tissues throughout the body [1]. The disease predominantly affects the lungs, skin, and lymph nodes; however, its diverse clinical manifestations can pose diagnostic dilemmas, particularly when atypical presentations occur. The neurological manifestations of sarcoidosis are diverse and can affect cranial nerves and the peripheral and central nervous systems [2]. Some neurological manifestations include cranial neuropathies, peripheral neuropathy, aseptic meningitis, optic neuritis, vasculitis, and myelopathy [3]. Longitudinally extensive transverse myelitis (LETM) is an uncommon disorder of the spine with significant inflammation, which stands out among the rare neurological complications of sarcoidosis. Unlike the more common pulmonary involvement, sarcoidosis-associated LETM presents distinctive challenges due to its varied clinical expression. LETM is characterized by extensive inflammation across the spinal cord, leading to rapid-onset motor, sensory, and autonomic deficits [4]. We present a case of LETM in a female who presented with neurological manifestations as an initial manifestation of sarcoidosis.

## Case presentation

A 62-year-old female without significant medical history presented to the emergency department with progressive bilateral lower extremity weakness for the last 17 days. Onset was gradual and progressive, with no aggravating or relieving factors associated with sensory disturbance and urinary retention for the last nine days. She had no history of recent infection, fever, travel history, or trauma. She was not using any medication. She did not report smoking, alcohol, or illicit drug abuse and had no family history of a similar disease.

**Figure 1:**
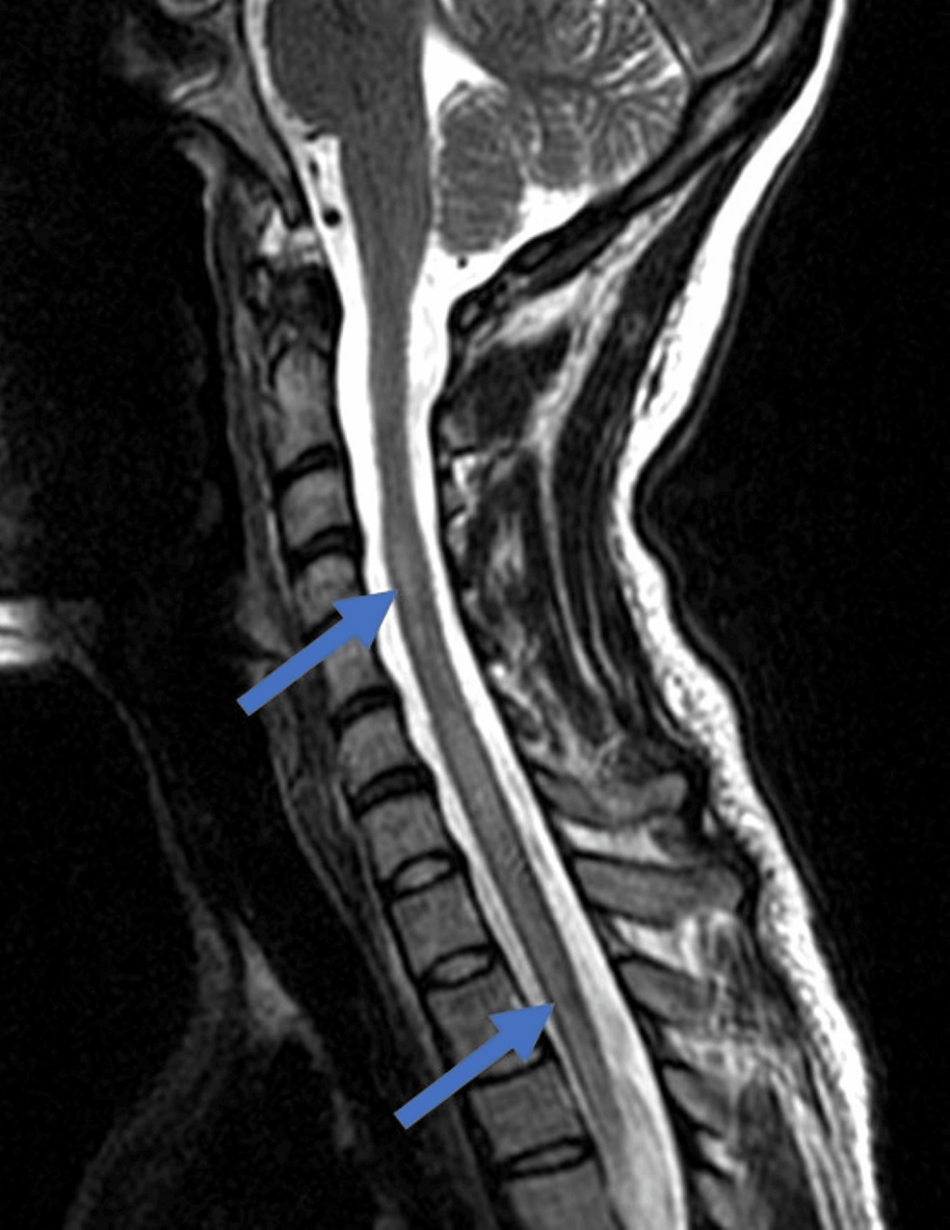
T2-weighted spine MRI demonstrating hyperintense signals in the cervical and thoracic spine (blue arrows). MRI: magnetic resonance imaging

**Figure 2:**
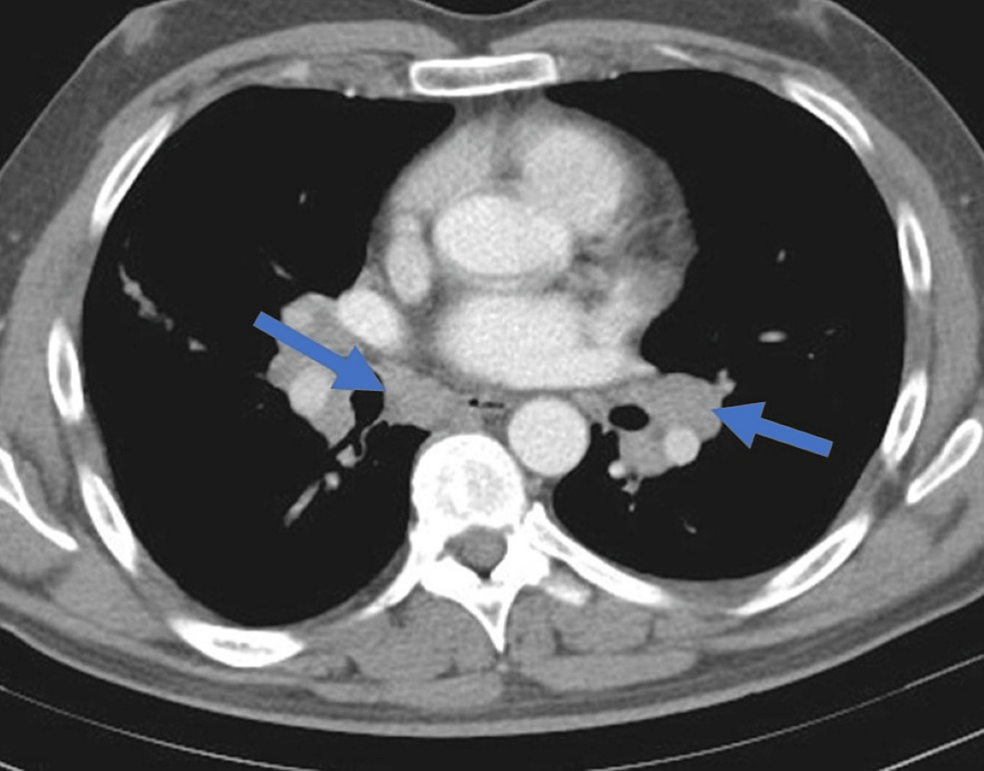
Chest computed tomography revealing bilateral hilar lymphadenopathy (blue arrows).

On initial evaluation, she was hemodynamically stable and oriented to time, place, and person. On neurological examination, she had bilateral lower extremity weakness, graded as 4/5 on the Medical Research Council (MRC) scale. The weakness was more pronounced in the lower limbs, affecting both proximal and distal muscle groups, with absent reflexes. She also underlined bilateral hypoesthesia. Cranial nerves were intact, and systemic examination was normal. Initial laboratory investigations were within normal limits, including a complete blood picture, basic metabolic profile, and inflammatory markers. She underwent cerebrospinal fluid analysis, which revealed elevated protein and white cell count, suggesting inflammation. Brain magnetic resonance imaging (MRI) was normal, and spine MRI demonstrated an extensive T2 hyperintense lesion from C4 to T4, characteristics of LETM (Figure 1). Further imaging studies, including brain MRI, were unremarkable. Chest computed tomography (CT) revealed hilar lymphadenopathy and mediastinal adenopathy (Figure 2). Autoimmune and infectious workups were negative, including tests for aquaporin-4 antibodies and myelin oligodendrocyte glycoprotein antibodies. Nerve conduction studies and electromyography were consistent with a myelopathic process.

**Figure 3:**
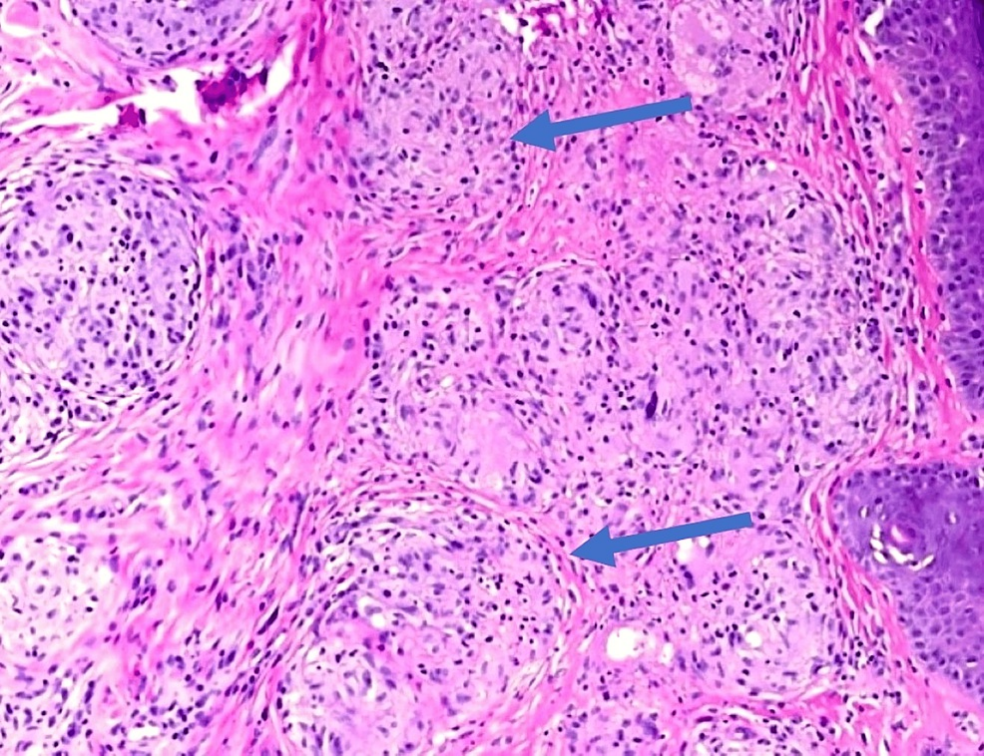
Lymph node biopsy revealing clusters of epithelioid cells with multinucleated giant cells consistent with non-caseating granulomas. Stains: hematoxylin and eosin (magnification: 40×).

Given the clinical picture and imaging findings, the patient underwent a thoracic spine biopsy, which revealed non-caseating granulomas consistent with sarcoidosis (Figure 3). Systemic evaluation, including ophthalmologic examination, gallium scintigraphy, and pulmonary function tests, did not show evidence of involvement in other organs. The patient was diagnosed with sarcoidosis-associated LETM. She was initiated on high-dose corticosteroids, resulting in a gradual improvement of neurological symptoms. Subsequently, she was commenced on methotrexate. She underwent a follow-up MRI of the spine after three weeks, which yielded a reduction in the size and an enhancement of the spinal lesion. Over the next several months, the patient's neurological deficits continued to improve, and she regained near-normal function.

## Discussion

LETM, being a life-threatening condition, has a diverse etiology, and sarcoidosis is the diagnosis of exclusion (Table 1) [5,6]. The literature on sarcoidosis-induced LETM is sparse but gradually expanding. Existing case reports and small case series underscore the diverse clinical spectrum of neurological involvement in sarcoidosis, with LETM emerging as a rare but significant manifestation. Only a limited number of reports have documented cases where LETM was the primary presenting feature, emphasizing the importance of considering sarcoidosis in the differential diagnosis of myelopathic syndromes [7]. The variability in clinical presentations, treatment responses, and long-term outcomes highlights the need for further research to elucidate the underlying mechanisms and optimal management strategies. Wang and Li reported an analysis of seven cases of sarcoidosis-induced LETM with a median age of onset of 49.1 years old. The angiotensin-converting enzyme (ACE) level was raised in four patients. LETM was diagnosed with MRI in all patients, and the diagnosis of sarcoidosis was confirmed using chest imaging. All the patients reported improvements after the commencement of steroids and immunosuppressive management [8]. Cicia et al. underlined a case of sarcoidosis-induced LETM in a 60-year-old male with no systemic manifestations, and his condition improved after starting immunosuppressive therapy [9]. Rodrigues et al. also reported a case of LETM as an initial manifestation of sarcoidosis in a 53-year-old male. After a detailed imaging and mediastinal lymph node biopsy, he was diagnosed with sarcoidosis-induced LETM [10]. Scott et al. also reported two cases of LETM induced by sarcoidosis. Both patients presented with neurological manifestations and were diagnosed with LETM on MRI imaging, and biopsy findings confirmed non-caseating granulomas [3].

**Table 1:** Etiology of transverse myelitis Source: [5,6]

Categories	Cause
Infections	Enteroviruses
	West Nile virus
	Herpes virus
	Zika virus
	Bacterial skin infections
	Mycoplasma bacterial pneumonia
Systemic inflammatory autoimmune diseases	Systemic lupus erythematous disease
	Sarcoidosis
	Scleroderma
	Rheumatoid arthritis
	Mixed connective tissue disease
	Behcet disease
Central nervous system diseases	Multiple sclerosis
	Neuromyelitis optica spectrum disorder
	Acute disseminated encephalomyelitis

The pathophysiology of sarcoidosis-induced LETM remains incompletely understood. Sarcoidosis is characterized by the formation of non-caseating granulomas, which can lead to inflammation and subsequent myelitis when present in the spinal cord [11]. Immunological mechanisms, including T-cell dysregulation and cytokine release, likely contribute to the formation of granulomas and the inflammatory cascade within the spinal cord [12]. The heterogeneity in clinical presentations suggests that multiple pathogenic pathways may be involved, necessitating further investigation into the immunological and genetic factors contributing to LETM in sarcoidosis.

Diagnosing sarcoidosis-induced LETM requires a comprehensive approach. Imaging studies, such as MRI of the spine, are crucial in visualizing the characteristic longitudinally extensive lesion [13]. Additionally, a thorough evaluation of cerebrospinal fluid, including cell count, protein, and oligoclonal bands, aids in confirming the inflammatory nature of the myelitis [9]. Systemic investigations, such as chest imaging and ophthalmologic examination, are essential to identify the multiorgan involvement characteristic of sarcoidosis. Despite these diagnostic modalities, the challenge lies in distinguishing sarcoidosis-induced LETM from other causes of inflammatory myelopathies, necessitating careful exclusion of alternative etiologies [14].

Managing sarcoidosis-induced LETM involves immunosuppressive therapy to modulate the aberrant immune response. High-dose corticosteroids, such as prednisone, are commonly initiated as first-line treatment to mitigate inflammation [10]. For cases requiring long-term immunosuppression or refractory to corticosteroids, steroid-sparing agents such as infliximab, methotrexate, azathioprine, or mycophenolate mofetil may be considered [15]. The close monitoring of treatment response and potential side effects is paramount. Rehabilitation strategies, including physical and occupational therapy, are crucial in optimizing functional outcomes, particularly in cases with residual neurological deficits [14,15].

## Conclusions

Although rare, neurosarcoidosis should be considered in the differentials of LETM, even in the absence of previously diagnosed sarcoidosis. The rarity of sarcoidosis-induced LETM underscores the need for larger prospective studies to characterize better the clinical course, treatment responses, and prognostic factors associated with this manifestation. Investigating the immunogenetic basis of sarcoidosis, particularly in cases with neurological involvement, may provide insights into disease mechanisms and potential therapeutic targets. Collaborative efforts between neurologists, rheumatologists, and other specialists are crucial for a multidisciplinary approach to understanding and managing this uncommon yet impactful facet of sarcoidosis.
